# P-943. Evaluation of a broad-spectrum antibiotic monitoring tool across a large academic hospital system

**DOI:** 10.1093/ofid/ofaf695.1146

**Published:** 2026-01-11

**Authors:** Natalie Tucker, Dayna McManus, Matthew W Davis, Pegah Russo, Indigo Moss, Jeffrey E Topal, Victoria Fal, Evita Santos, Alexander Michno, Emily Minella

**Affiliations:** Yale New Haven Hospital, New Haven, CT; Yale New Haven Hospital, New Haven, CT; Yale New Haven Hospital, New Haven, CT; Lawrence + Memorial Hospital, New London, Connecticut; Yale New Haven Health, New Haven, Connecticut; Yale New Haven HospitalYale University School of Medicine,, New Haven, Connecticut; Lawrence + Memorial Hospital, New London, Connecticut; Yale New Haven Health, New Haven, Connecticut; Yale New Haven Health, New Haven, Connecticut; Lawrence + Memorial Hospital, New London, Connecticut

## Abstract

**Background:**

Broad spectrum antibiotic (BSA) use within the Yale New Haven Health System (YNHHS) is evaluated daily as part of pharmacist workflow. To prompt evaluation of these antibiotics, the electronic medical record (EMR) alerts pharmacists and providers based on use greater than 48 hours and all expiring/expired BSA orders placed. Pharmacists are expected to document according to these alerts using a template designed to determine therapy appropriateness and encourage antibiotic stewardship interventions. The objective of this research was to assess overall compliance with and quality of documentation to better understand antibiotic stewardship challenges and identify opportunities for improvement.

**Methods:**

This was a quality improvement assessment with same-day, concurrent medical chart review at all five hospitals within YNHHS. Patients admitted from October 1, 2024, through November 15, 2024 who had orders for BSA (ceftriaxone, ceftazidime, cefepime, or piperacillin-tazobactam) greater than 48 hours and/or had expiring/expired antibiotic orders were included. To monitor overall compliance, evaluation of the total number of alerts per day and number of alerts documented were assessed three times per week. To assess the quality of documentation, 10 random patient charts were reviewed each week at each site for appropriate utilization of the prespecified template. Data collection obtained from the EMR included patient demographics, current antibiotics, indication, day of therapy, culture results, pharmacist assessment, intervention, and follow-up.

**Results:**

Two hundred patient charts were reviewed. Overall compliance with the specific note template was 31%. When the template was used, ten interventions (16%) were made to de-escalate antibiotics, and five interventions (8%) were made to discontinue antibiotics. Forty-seven recommendations (76%) were made to continue antibiotics as they were deemed appropriate or additional information was needed prior to intervening.

**Conclusion:**

Compliance with the pharmacist BSA note template was low. The combined effort of providers and pharmacists is needed to promote optimal antimicrobial stewardship efforts. Meeting with frontline pharmacists to adjust the note may increase compliance and therefore number of interventions.

**Disclosures:**

All Authors: No reported disclosures

